# 25 years of research and regulation: Is nanotechnology safe to commercialize?

**DOI:** 10.3389/ftox.2025.1629813

**Published:** 2025-07-10

**Authors:** Kirsten Rasmussen, Phil Sayre, Andrej Kobe, Mar Gonzalez, Hubert Rauscher

**Affiliations:** ^1^ European Commission, Joint Research Centre (JRC), Ispra, Italy; ^2^ nanoRisk Analytics LLC, Auburn, CA, United States; ^3^ European Commission, Directorate-General for Environment, Brussels, Belgium; ^4^ Organisation for Economic Co-Operation and Development (OECD), Environment Directorate, Paris, France

**Keywords:** safety of nanomaterials, nanomaterial definition, nanomaterials legislation, nanomaterials governance, OECD test guidelines for nanomaterials, FAIR data

## Abstract

This paper examines the global communities’ regulatory and scientific advancements in nanotechnology and nanomaterials since 2000. It explores some similarities and differences in nanomaterial safety compared to general chemical safety. The paper provides an overview of the encountered challenges and how far they have been resolved, as well as information on how different countries’ legislators have addressed nanomaterials, including safety assessment in (new) legislation. Challenges arose due to the unique physico-chemical properties of some nanomaterials and included the lack of i) a regulatory definition, ii) applicable regulatory test methods, including methods for physico-chemical characterization and for ecotoxicological effects, as well as sample preparation and dosimetry, iii) assessment and modelling of human, especially occupational, and environmental exposure to nanomaterials, iv) quantification of nanomaterial in complex media, v) systems for collecting the data generated and ensuring FAIR (Findable, Accessible, Interoperable and Re-usable) and quality data, vi) reference nanomaterials, and vii) a frame for nanotechnology governance. The paper highlights the role of the Organisation for Economic Co-operation and Development (OECD) in building a global, regulatory understanding of nanotechnology and nanomaterials, as well as the OECD’s achievements of developing nano-specific test guidelines. The paper identifies areas, such as alternative test methods, availability of reference nanomaterials, comparable data and FAIR data, analytical tools for quantifying nanomaterials in (complex) matrices that are still under-addressed. It gives a wider perspective of Governance of Advanced Materials including nanomaterials, also illustrated by carbon nanotubes used in batteries for electric vehicles, to also aid their commercialization. In the EU, the policy context is moving towards a holistic governance approach embracing sustainability dimensions.

## 1 Introduction

About year 2000 nanotechnology and nanomaterials were designated as key enabling technologies, reinforcing their position in groundbreaking technological development and research in widely diverse sectors such as electronics, energy, medicine, construction, food, textile, and transport. “Nanotechnology” describes those areas of science and engineering that utilize phenomena that take place at dimensions in the nanometer scale in the design, characterization, production and application of materials, structures, devices and systems^1^. The increasing application of nanomaterials and nanotechnology has prompted regulatory science research, e.g., into identification of potential adverse effects (Oberdörster et al., 2005), to enable the safety assessment of nanomaterials intended for commercialization. Thus, for example the European Union funded a plethora of research projects under several of its multi-annual EU research framework programs to investigate safety and other aspects of nanomaterials (Farcal et al., 2023).

An initial challenge was to define “nanomaterial”. This has converged towards an agreement that such materials are “at the nanoscale” which generally refers to the size range of 1 nm–100 nm (Rasmussen et al., 2024). Consequently, the attribute “nano” is often regarded as solely describing size (e.g., ISO, 2023; European Commission, 2022a), though certain legal definitions and descriptions may also require a nanomaterial to exhibit “additional properties” (e.g., Malaysia, 2018). The particle size is indeed the only common feature shared by all nanomaterials, though it may not fully capture the potential for novel risks. It is recognized that at the lower end of the size range, there may indeed be truly nano-related effects. These will vary with chemical identity. A hard upper size cut-off for the occurrence of such effects, required for regulatory certainty, may not always reflect the full risk-continuum. Such risks might arise even at larger sizes; e.g., the pharmaceutical industry uses an upper cut-off of 1,000 nm (Mohanraj and Chen, 2006).

Assessing the safety of nanomaterials and nanotechnology is further complicated by their rapid evolution. Nanotechnologies develop across multiple overlapping and increasingly advanced generations, driven by the convergence of different science and engineering disciplines (Roco, 2018; ECHA 2019b). Increasingly complex materials incorporating several nanoscale components and/or other larger components add complexity beyond size-related considerations of a single particle (Hunt et al., 2025; Swart et al., 2025). This development has created a need for (new) assessment tools that can better address the properties and possible ecotoxicological effects, fate, and risks of increasingly complex nanomaterials. A prerequisite for determining environmental fate and biodistribution is the ability to detect and reliably quantify nanomaterials in both environmental and *in vitro* media and in organismal tissues and fluids (DeLoid et al., 2017; Reagen and Zhao, 2022). Additionally, it is a challenge to legislate for such complex structures, which may fit several legal definitions. For example depending on the context, the same multicomponent nanomaterial can fit “substance”, “mixture” and “article” (ECHA et al., 2019a; Hunt et al., 2025) under the EU (European Union) REACH Regulation (Registration, Evaluation, Authorisation and Restriction of Chemicals; European Parliament and Council, 2006).

Initially, nanomaterials were regarded as a new class of materials that possibly carried novel risks related to their particle size. One challenge for nanomaterials is that in addition to their chemical properties, nanomaterials may have safety relevant physical properties associated with their particles-chemical duality, which complicates several aspects of their safety assessment. For example, established test methods for identifying possible hazards may need to be adjusted or at least confirmed for nanomaterials. Moreover, exposure assessment has both quantitative and qualitative challenges, as well as considerations of what the organisms and environment are actually exposed to as nanoparticles in a medium are surrounded by a corona (Behzadi et al., 2017; Lundqvist and Cedervall, 2020) and/or are subject to changes in physico-chemical properties over time, depending on the medium (Klaper, 2020). Furthermore, the fate models need to consider particle behavior. The available analytical methods for detection and quantification of nanomaterials may be inadequate, due to challenges such as the extraction of nanomaterials from complex media. Moreover, nanoparticle behavior in a medium is governed by kinetic processes. Hence, the water-octanol partition coefficient, *K*_ow_ is meaningless for nanoparticles, and fate and bioaccumulation modelling for nanomaterials cannot rely on equilibrium partitioning, which is the default approach.

In parallel to scientific considerations, legislators considered whether nanomaterials were adequately addressed by chemicals’ legislation, or if amended, or additional, legislation would be needed. Furthermore, the adequacy of regulatory methods for safety assessment was considered, including hazard and exposure assessment, and risk characterization. Additionally, the importance of data and especially FAIR (Findable, Accessible, Interoperable and Re-usable) data (Jeliazkova et al., 2021) and complete metadata associated to any measurement result on nanomaterials was stressed (Comandella et al., 2020).

In 2006, the OECD (Organisation for Economic Co-operation and Development) established the Working Party on Manufactured Nanomaterials (WPMN) to provide a global forum for nanosafety discussions (Rasmussen et al., 2016; 2018; 2019). The OECD was identified as one of the key bodies to address nanotechnology governance issues (Morris et al., 2011), combining identification, assessment, management, evaluation and communication, considering the way decisions are taken by the different actors (researchers, industry, policymakers, regulators, *etc.*). The WPMN scrutinizes the methodologies available to assess the safety of nanomaterials, including the human and environmental exposure (OECD, 2021a; b; c; d). Additionally, the OECD Council issued a Recommendation on the Safety Testing and Assessment of Manufactured Nanomaterials (OECD, 2013; 2017c), which aims to align the safety testing and assessment of nanomaterials with that of chemicals, notably, on Mutual Acceptance of Data in the assessment of chemicals. This Recommendation expresses that existing legal frameworks, adapted to nanomaterials, can be used, and its Annex lists tools for testing and assessment (OECD, 2009b; OECD, 2012; OECD, 2015; OECD, 2022a), which should be used in conjunction with the appropriately adapted OECD TGs that take into account the specific properties of nanomaterials. Another challenge when testing nanomaterials is that for the same chemical composition a multitude of different nanomaterials may exist, potentially having diverse properties and risks, which challenge some default regulatory approaches.

Alternative methods are gaining importance as regulatory testing of chemicals moves away from animal testing, leading to a need for reliable and relevant *in vitro* and other non-animal methods (Browne et al., 2019). This type of testing of nanomaterials presents its own set of challenges, e.g., cells are exposed to particles, not to uniformly distributed dissolved chemicals, and available methods, let alone regulatory methods, are generally not yet validated for nanomaterials. Neither are methods such as QSARs (quantitative structure activity relationships) and Grouping and Read Across, and it is a challenge to identify datasets that would allow such a validation. Another challenge is the regulatory recognition of such methods.

As the research community started to address these challenges, some scientists predicted the possibility of nanospecific ecotoxicological effects (Maynard, 2012; CIEL and Öko-Institut, 2015). Others (e.g., Donaldson and Poland, 2013; Krug, 2014) noted that while nanomaterials seemingly presented a need for unique considerations, many of the issues suggested as relevant for nanomaterials had already been identified for other types of chemicals. Contrary to larger particles, certain nanoparticles may translocate within organisms, i.e., cross both primary (e.g., air-blood/lungs; intestinal) and secondary (i.e., barriers that prevent tissues from interacting with the contents of circulating blood, e.g., the placenta) biological barriers (Cary and Stapleton, 2023). Thus, translocation may lead to adverse effects in a different organ than the point (organ) of entry into the organism. For example, translocation from the olfactory bulb to the brain has been documented (Kreyling, 2016) or from the lungs to systemic circulation, possibly being the underlying factor for their toxicity to multiple organs (Raftis and Miller, 2019). Another aspect is the comparative toxicity of metal and metal oxide nanoparticles and their larger counterparts. Test results have demonstrated that sometimes the nanoparticles can exhibit a higher toxicity (e.g., Miao et al., 2024; Zhang et al., 2016; Mortimer et al., 2010; Kasemets et al., 2009; Heinlaan et al., 2008; Pan et al., 2007) though this is not always the case (Ali, 2023).

As outlined above, addressing the safety of nanomaterials in a legal context is a challenge compared to general chemicals. One response, filling some knowledge gaps, was the EU funded research projects under several of the multi-annual EU research framework programs to investigate safety and other aspects of nanomaterials (Farcal et al., 2023). These projects collaborated through the NanoSafetyCluster (NSC) (https://nsc-community.eu/).

This paper reviews how nanomaterial-specific regulatory challenges have been identified and addressed in science, their current (partial) solutions, and issues to be addressed in the future globally within the OECD, and by regional legislators, especially in the EU and United States. It describes some of the milestones and knowledge achievements in gaining deeper insights into nanomaterial safety assessment, and information is grouped into the areas that have been further developed for evaluating the safety of nanomaterials. While recognizing their importance, the paper does not address fields such as medicines based on nanomaterials or nanotechnology, nor nanoplastics. Neither does the paper address the work of standardization bodies.

## 2 Terminology and regulatory definition of nanomaterial

Common terminology is essential for any meaningful discussion, and for nanotechnologies ISO’s (International Organization for Standardization) vocabulary series aids technical discussions. A global discussion of the definition of “nanomaterial” took place within the OECD and within ISO, and some of the first issues agreed on were that in science “nano” is a prefix for one billionth (10^–9^), and that the nanoscale is the scale from (approximately) 1 nm–100 nm. ISO published a definition of *nano-object* and other core terms (ISO, 2010; 2023). The OECD agreed upon a working description of “nanomaterial” (Loevestam et al., 2010) to frame the discussions within the OECD WPMN.

**FIGURE 1:**
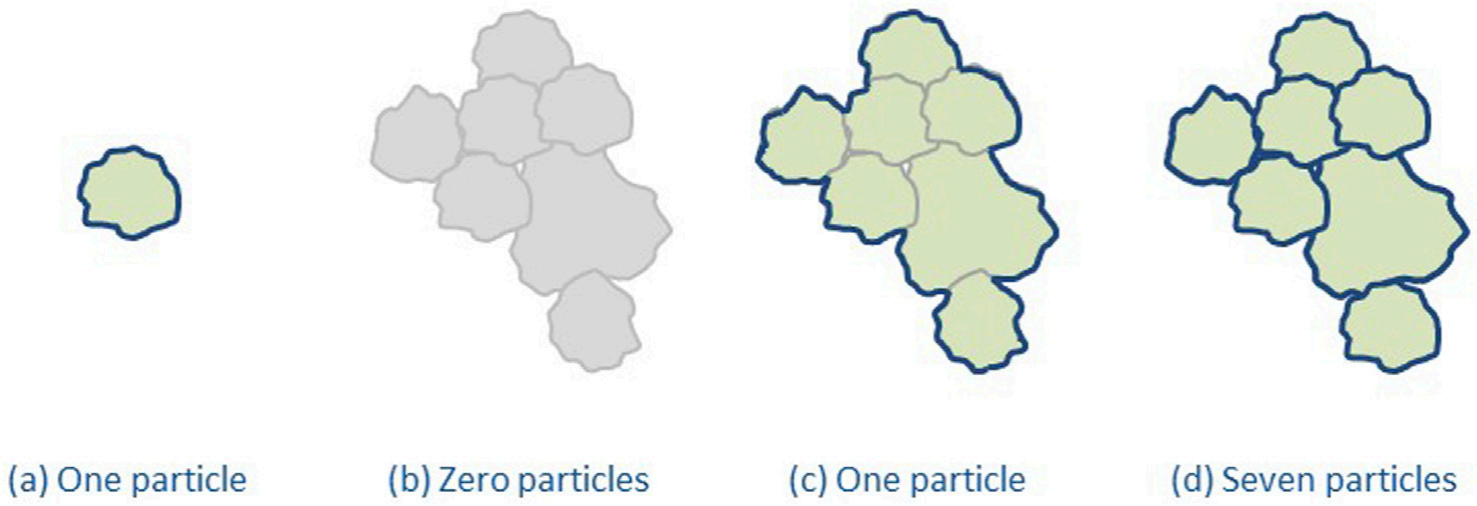
Consideration of agglomerates and aggregates in nanomaterial definitions. **(a)** is an individual unbound particle, **(b)** illustrates that some definitions disregard agglomerates and aggregates, **(c)** illustrates that other definitions count them as one particle, and finally **(d)** illustrate that yet other definitions count each particle within them (based on Bresch et al., 2022/Rasmussen et al., 2024).

**FIGURE 2:**
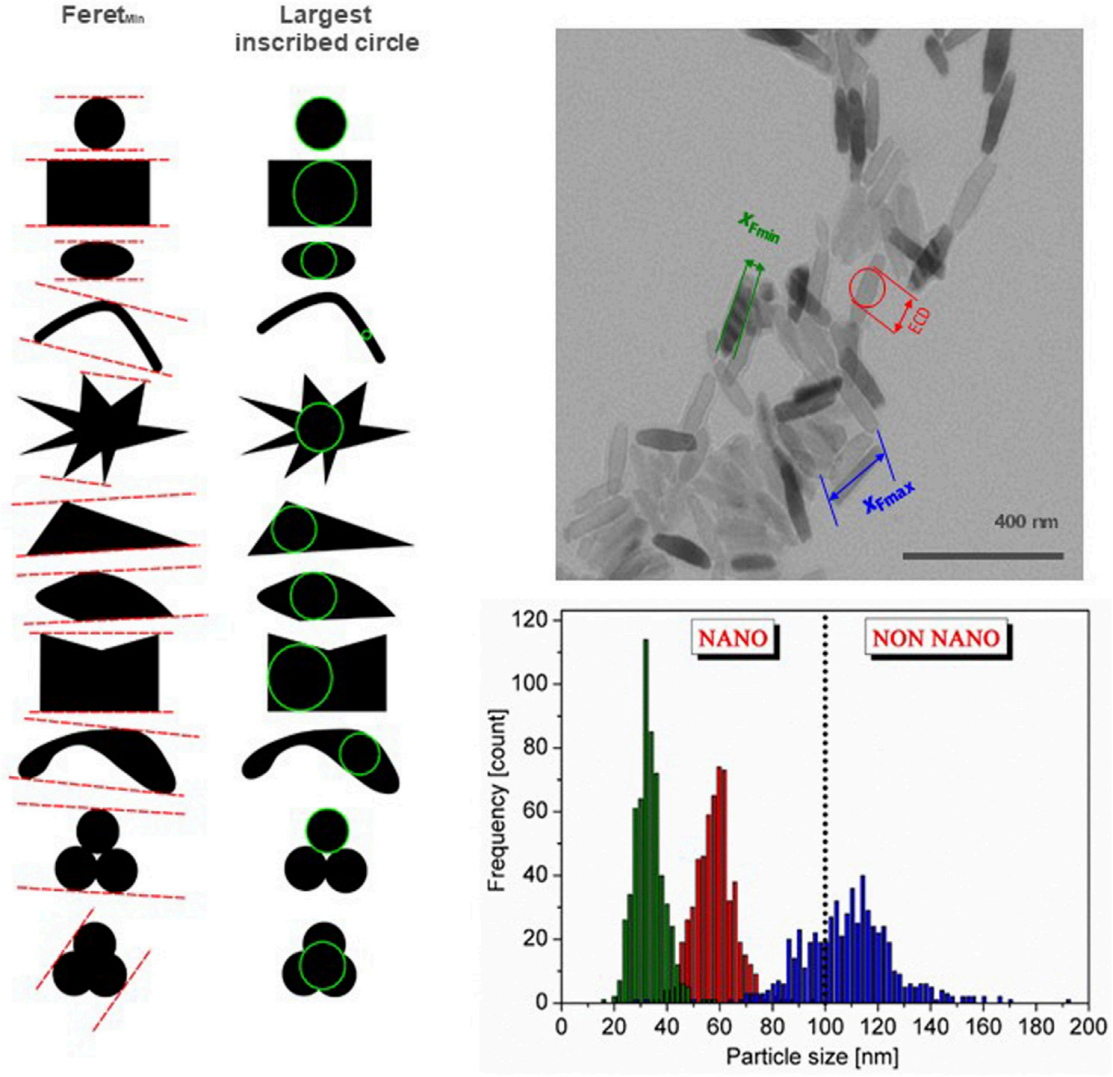
Illustration of representation of size by minimum Feret diameter and the largest inscribed circle for the same particle shape (left). The right side illustrates the particle size distribution for the same material represented by three different external dimensions, the Feret_min_ and Feret_max_ and the equivalent circular diameter (ECD) (top, right), and the resulting particle size distribution (bottom, right). Depending on the chosen measurands the material can be a nanomaterial or not a nanomaterial based on particle number size distribution. After Rauscher et al., 2019a; b ^©^ European Commission.

The agreement of what the nanoscale is helped to define *nanomaterial* in a legal context, which ensures transparency both for regulators and Industry, and several countries/regions have developed regulatory definitions (Rasmussen et al., 2024). However, a number of uncertainties for regulatory definitions of *nanomaterial* still remain, as, e.g., most particulate materials are a mixture of particles at the nanoscale and larger ones. Additionally, particles often form agglomerates and aggregates that are larger than 100 nm. To be implementable, a regulatory nanomaterial definition needs to define a threshold for the amount of nanosized particles necessary for a material to be a nanomaterial and the metrics to measure this (e.g., particle number-based or mass fraction). Such a threshold means that there will also be non-nanomaterials on the market containing a fraction of material at the nanoscale. Furthermore, the way agglomerates and aggregates are taken into account needs to be stated, see Figure 1. It should be noted that the measured particle size depends on the measurement method used (Mech et al., 2020) and the measurand chosen to describe the size, see Figure 2 (Rauscher et al., 2023). Furthermore, regulatory definitions may refer to the origin of the nanomaterial, i.e., whether it is manufactured/engineered, incidental or natural.

A tailored regulatory definition of *nanomaterial* should ensure that any possible legal gaps are minimized. As illustrated here and noted in Sayre et al. (2017), the differences in nanomaterial definitions for regulatory and research purposes affect which nanomaterials, or nano-enabled products, are subject to regulatory review, and what sort of research and guidance for industry is pursued by regulatory bodies.

The U.S. National Nanotechnology Initiative (NNI) defined nanotechnology, including a definition of “matter at the nanoscale”, (US NNI, 2011; 2024), noting that “… Matter can exhibit unusual physical, chemical, and biological properties at the nanoscale…” This definition was intended to guide research into developing materials in a particular size range, which also possessed unique properties, and from it, at least two different regulatory approaches evolved in the United States. Both the US EPA (Environmental Protection Agency) and the FDA (U.S. Food and Drug Administration) decided early on not to define the term nanomaterial. The EPA rather triggers its regulatory review of chemical substances when they have structures with dimensions at the nanoscale, and notes, in line with the NNI definition, that chemical substances at the nanoscale may behave differently than conventional chemicals under specific conditions. The FDA agreed that the nanoscale range is “approximately 1 nm–100 nm” and noted that according to the FDA definition a nanotechnology product behaves differently from a conventionally manufactured product. However, the FDA extended the size range for examining FDA-regulated products to larger scales up to 1,000 nm (US FDA, 2014). The EU defined nanomaterial early on for sectoral legislation and currently several regulatory definitions exist (Rasmussen et al., 2024). The EU has adopted a recommendation for a definition of nanomaterial applicable across legislation (European Commission, 2011; 2022a) and works on its implementation. According to this definition, the building blocks (“constituent particles”) of agglomerates (and aggregates) are measured and counted.

Some regional regulatory definitions require that a nanomaterial has “additional properties” compared to its corresponding bulk material (e.g., US FDA, 2024; Malaysia, 2018). Some regions (e.g., China, 2011; 2020) define nanomaterial in their legislation by referring to the ISO definition (ISO, 2023), which does not include a threshold for the amount of nanosized particles necessary for a material to be a nanomaterial.

Hence, even with the internationally converging agreement that the nanoscale is 1 nm–100 nm, there are important differences between countries’ legal definition(s) of “nanomaterial”, which should be known and recognized in order to improve transparency.

## 3 Regulatory testing of nanomaterials

Regulatory testing for identifying hazardous properties of chemicals/substances is performed according to OECD Test Guidelines (TGs) whenever possible. TGs are developed in the OECD Test Guidelines Programme (TGP) through a consensus process. They are fundamental to OECD’s system of Mutual Acceptance of Data (MAD) (OECD, 1981), which is a legally binding instrument to facilitate the international acceptance of information for the regulatory safety assessment of chemicals. Tests performed according to TGs, and following the principles of Good Laboratory Practice (GLP) (OECD GLP, 2025b.), are recognized in countries adhering to MAD. The OECD also develops Guidance Documents (GDs) on testing, which, while not falling under MAD, nevertheless reflect an agreement on best available procedures. The OECD GD 34 (OECD, 2005) gives guidance on the validation and international acceptance of test methods for hazard assessment. It must be ensured that TGs are relevant, reliable and adequate for testing nanomaterials. Hence, the WPMN oversees preparation of nanomaterial-relevant method proposals for the TGP. The WPMN supervised the testing of 11 different types of nanomaterials (Rasmussen et al., 2016), which investigated relevant endpoints, the applicability of TGs to nanomaterials (OECD, 2009b), and practical aspects of testing such as preparing test samples (OECD, 2012). The outcomes of this testing and assessment program, combined with general progress in science, provided a basis for ensuring the applicability of TGs to nanomaterials and the further development of TGs and GDs relevant for nanomaterials, see Table 1. Additionally, the WPMN has evaluated tools and models used for assessing human (OECD 2021a; b; c) and environmental exposure to nanomaterials (OECD, 2021d).

**TABLE 1:** OECD TGs and GDs amended/developed to address nanomaterial-specific aspects, ongoing projects in the OECD TGP that are particularly relevant for nanomaterials as well as selected publications by the OECD WPMN.

New and updated test guidelines (TGs) and guidance documents (GDs) for nanomaterials
TG 124 Determination of the Volume Specific Surface Area of Manufactured Nanomaterials (OECD, 2022d)
TG 125 Nanomaterial Particle Size and Size Distribution of Nanomaterials (OECD, 2023b)
TG 126 Determination of the Hydrophobicity Index of Nanomaterials Through an Affinity Measurement (OECD, 2023c)
TG 318 Dispersion Stability of Nanomaterials in Simulated Environmental Media (OECD, 2017c)
TG 412 Subacute Inhalation Toxicity: 28-Day Study (OECD, 2018e)
TG 413 Subchronic Inhalation Toxicity: 90-day Study (OECD, 2018f)
TG 444A Test Guideline on *In Vitro* Immunotoxicity: IL-2 Luc Assay (OECD, 2023d)
GD 39 Inhalation Toxicity Studies. Series on Testing and Assessment No. 39 (Second Edition) (OECD, 2018c)
GD 286 Guidance Document on Good *In Vitro* Method Practices (GIVIMP) (OECD, 2018b)
GD 317 Guidance Document on Aquatic and Sediment Toxicological Testing of Nanomaterials (OECD, 2021e)
GD 318 Guidance Document for the Testing of Dissolution and Dispersion Stability of Nanomaterials and the Use of the Data for Further Environmental Testing and Assessment Strategies (OECD, 2020b)
GD 342 Guidance Document on Testing Nanomaterials Using OECD TG No. 312 “Leaching In Soil Columns” (OECD, 2021f)
Study Report on a Test for Removal in Wastewater Treatment Plants of Gold Manufactured Nanomaterial (MN): Activated Sludge Sorption Isotherm (OECD, 2021g)
Study Report and Preliminary Guidance on the Adaptation of the *In Vitro* micronucleus assay (OECD TG 487) for Testing of Manufactured Nanomaterials (OECD, 2022b)
Study Report on Applicability of the key event-based TG 442D for *in vitro* skin sensitisation testing of nano-materials (OECD, 2023a)
A tiered approach for reliable bioaccumulation assessment of manufactured nanomaterials in environmental organisms minimising use of higher tier vertebrate tests (OECD, 2024b)
New GD on assessing the apparent accumulation potential for nanomaterials (2025), in print
Updated Guidance Document on Sediment and Aquatic Toxicity Testing of Nanomaterials (2025) (guidance for TG 201, TG 202 and TG 203), in print

Ongoing projects in the Test Guidelines Programme that are particularly relevant for nanomaterials
Determination of solubility and dissolution rate of nanomaterials in water and relevant synthetic biologically media (Project 1.5)
Guidance Document on Identification and quantification of the surface chemistry and coatings on nano- and microscale materials (Project 1.6)
TG on Determination of the Dustiness of Manufactured Nanomaterials (Project 1.8)
New Guidance document on the determination of concentrations of nanoparticles in biological samples for (eco)toxicity studies (Project 1.10)
Revision of GD 317 on Aquatic Toxicity Testing of Nanomaterials (Project 2.71)
New TG on dissolution rate of nanomaterials in aquatic environment (Project 3.10)
Guidance Document Environmental abiotic transformation of nanomaterials (Project 3.16)
New Guidance Document on toxicokinetics to accommodate testing of nano-particles (Project 4.146)
New GD on Integrated Approaches to Testing and Assessment (IATA) for intestinal fate of orally ingested nanomaterials (Project 4.158)
Validation of the *In Vitro* Micronucleus assay (TG 487) for Engineered Nanomaterials (Project 4.174)

Selected publications by the WPMN
Guidance on Sample Preparation and Dosimetry for the Safety testing of Manufactured Nanomaterials (OECD, 2012); currently under revision
Important Issues on Risk Assessment of Manufactured Nanomaterials (OECD, 2022a)
Guiding Principles for Measurements and Reporting for Nanomaterials: Physical Chemical Parameters (https://doi.org/10.1787/70c84148-en)
Physical-Chemical Decision Framework to inform Decisions for Risk Assessment of Manufactured Nanomaterials (https://doi.org/10.1787/1fbbaf8c-en)

Furthermore, policymakers have monitored and analyzed the gaps and progress (e.g., SCENIHR, 2006; 2007a; b, 2009; US NNI, 2011; 2024; European Commission, 2012; 2018) of information needs for assessing the safety of nanomaterials and suggestions for methodological gap-filling or updates, such as the need to develop new TGs or adapt existing ones. The regulatory testing of nanomaterials presents a specific challenge, as more than one dataset may be needed per chemical composition as properties and fate depend on nanomaterials’ physical form as well.

Table 1 lists both published nanomaterial-specific TGs and GDs and the ones currently under development or adaptation. The latter include three new nanomaterial-specific methods that relate to the environmental fate of nanomaterials. Two methods address biological fate of nanomaterials: mammalian toxicokinetics in general, and an intestinal fate-specific IATA (Integrated Approaches to Testing and Assessment). Finally, there are three GDs for physico-chemical characterization of nanomaterials (on quantification of nanomaterial surface attributes and on the solubility and dissolution rate of nanomaterials in water-based media), and one new TG for determination of dustiness. Other TGs, which are not specifically adapted to nanomaterials, can be used for the assessment of some nanomaterials. For example, OECD TG 439 on *In Vitro* Skin Irritation: Reconstructed Human Epidermis Test Method (OECD, 2021h) and several alternative OECD TGs for skin sensitization (e.g., OECD, 2024a), accommodate the testing of powders. These TGs include precautions regarding their use, based on factors such as the solubility of the powders and their interactions with test reagents. Additionally, the WPMN is updating the Guidance on Sample Preparation and Dosimetry for the Safety testing of Manufactured Nanomaterials (OECD, 2012), see below; publication is expected in 2025.

### 3.1 Sample preparation and dispersion media, a cross-cutting issue

The Guidance on Sample Preparation and Dosimetry (OECD, 2012) is especially important, as the sample preparation is key to the application of test protocols to nanomaterials and achieving reproducible results and it needs to be controlled, consistent, relevant, and reliable. The sample preparation and dispersion in appropriate media is an issue raised consistently when testing nanomaterials (e.g., Jensen et al., 2011; Hartmann et al., 2015; DeLoid et al., 2017), as many nanomaterials consist of particles and are insoluble or sparingly soluble in water and other media used in ecotoxicological tests. Among the factors influencing nanoparticle dispersion stability are the particle concentration, the physico-chemical characteristics of the nanoparticles (e.g., surface charge) and of the dispersion medium (e.g., pH, ionic strength), concentration of other substances and particles in the dispersion (e.g., natural organic matter), and the dispersion preparation procedure (e.g., OECD, 2012; 2017c). Moreover, the nanoparticles’ physico-chemical properties, and their ecotoxicological effects are highly influenced by the interactions with the bio-physical and chemical surroundings in the media.

The behavior of nanomaterials might give rise to artefacts (i.e., incorrect test results) during ecotoxicology studies (Petersen et al., 2014; Petersen, 2015; Park et al., 2009; Murray et al., 2018). Thus, care should be taken to avoid that the sample preparation introduces artefacts. They can be, e.g., presence of toxic impurities, incorrect nanomaterial storage, ineffective dispersion of the nanomaterial in the test medium, direct interference with assay reagents, un-acknowledged indirect effects such as nutrient depletion during the assay, and lack of proper assessment of the nanomaterial uptake by organisms (Petersen et al., 2014). Moreover, the unique properties of nanomaterials combined with a lack of appropriate test methods can lead to inaccurate and non-reproducible results (Petersen, 2015).

### 3.2 Physico-chemical characterization

Size and size distribution is the first parameter to characterize for a known (particulate) material to understand whether it is a nanomaterial, and if so, a set of additional properties are also of interest.

#### 3.2.1 Measurement of size and size distribution

Nanomaterials are generally defined by the size of the particles of which they consist, i.e., particles having at least one external dimension at the nanoscale. Thus, the accurate measurement of the size of particles at the nanoscale is of paramount importance. It is by no means a simple task, and often assumptions idealizing the particle shape are needed and only indirect methods can be applied, i.e., the measurand needs to be converted into a size (Mech et al., 2020), e.g., the dynamic light scattering method measures scattered light intensity, which is converted into particle size and size distribution. Moreover, several factors influence the measured particle size and size distribution, e.g., the particle shape, the measurement method, the chosen measurand, and how agglomerates and aggregates are taken into account (Bresch et al., 2022). The OECD TG 125 (OECD, 2023b) contains a compendium of methods for particle size measurement at the nanoscale. Furthermore, the NanoDefine Methods Manual (Mech et al., 2020) presents a catalogue of size measurement methods at the nanoscale and outlines the advantages and limits of each method. Figure 2 illustrates some of the issues to consider when measuring the size of nanoparticles.

#### 3.2.2 Further physico-chemical parameters for nanomaterials

Several nanospecific physico-chemical endpoints should be measured to properly characterize nanomaterials (OECD, 2009a; Stefaniak et al., 2013). Considerations on physico-chemical characterization of nanomaterials has led to integration of such requirements in legislation. Thus, REACH (European Parliament and Council, 2006) requires information such as surface functionalization or treatment, shape, aspect ratio and other morphological characterization, and information on surface area. The importance given to surface functionalization or treatment is noteworthy, as the surface is what the particle’s environment “sees”. The information on physico-chemical properties is important for understanding the environmental fate and behavior and the toxicokinetics of nanomaterials, and thereby the exposure targets of the nanomaterial. Thus, e.g., if the nanomaterial is surface functionalized or of core-shell type, the surface composition differs significantly from the “average” chemical composition which thus could be insufficient information.

The TGP has developed three TGs for physico-chemical characterization of nanomaterials: TG 124 on Determination of the Volume Specific Surface Area of Manufactured Nanomaterials (OECD, 2022d), TG 125 on Nanomaterial Particle Size and Size Distribution of Nanomaterials (OECD, 2023b) and TG 126 on Determination of the Hydrophobicity Index of Nanomaterials through an Affinity Measurement (OECD, 2023c). Testing according to TG 124 and TG 125 lead to information that allows an understanding of whether the material investigated could be a nanomaterial. The hydrophobicity information of TG 126 can be used as a surrogate for the n-octanol/water partition coefficient, which is inapplicable to insoluble, particulate nanomaterials.

In addition to OECD’s methods for regulatory testing, ISO, and especially ISO/Technical Committee (TC) 229 on nanotechnologies, develops documentary standards relevant for testing nanomaterials. Importantly, the European Committee for Standardization (CEN)/TC 137 deals with Assessment of workplace exposure and has published several standards on dustiness (CEN, 2019b; b, c; d, e).

## 4 Exposure to nanoscale materials and their measurement in the environment, at the workplace, and in biological tissue

### 4.1 General considerations for exposure

For safety assessment, it is fundamental to establish the routes of exposure and quantify exposure in the medium or in organisms. The assessment of soluble nanomaterials follow the one for their non-nano counterparts. To assess potential toxicity of other nanoparticles it is crucial to understand whether they enter organisms and cells after exposure as it is the internal dose, not the nominal (applied) dose, that is most relevant for understanding the toxicity (Gardner and Kirkpatrick, 2005) and biological actions of the nanoparticles, even at sub-cellular level (Behzadi et al., 2017), and thus the one that should be quantified. Behzadi et al. (2017) describe in detail nanoparticle aspects and processes for cellular uptake of nanoparticles. Furthermore, nanomaterials are often functionalized and therefore studies using the pristine nanomaterial may not be relevant for assessing the toxicokinetic or environmental behavior of the nanomaterials used. The quantification of human and environmental exposure to nanomaterials continues to be a major challenge and below some issues are listed.

Mass-based metrics (e.g., µg/mL) are the basis for the current hazard ranking of chemicals, and eases comparisons to the potential toxicity of analogue chemicals. For nanomaterials, Oberdörster (1996) and Donaldson et al. (1998) indicate that when particle number and available surface area drive the mode of action of nanomaterials, mass-based dosimetry cannot represent dose–response relationships. A nanoparticle is a distinct entity and should not be treated as a collection of atoms or molecules, as this may lead to erroneous comparisons (Simkó et al., 2014). Additionally for comparison of different nanomaterials with the same chemical composition, or corresponding non-nano form(s), mass concentration may not be adequate, as particle size and specific surface area may play a main role in determining their toxicity. There are not yet any definitive conclusions on the best dose metric, and dose-effect behavior may be better expressed as a function of concentrations of surface (e.g., cm^2^ of particle surface/mL), deposited surface area (e.g., cm^2^ of deposited particle surface/cm^2^ of cell surface), or number (e.g., number of particles/mL).

In risk assessment, exposures to a chemical are considered relative to its potential hazard. Sometimes production volume is used as a surrogate for exposure or to estimate exposures to workers or the environment. These estimated exposures are based on factors such as engineering controls for a workplace exposure and prior monitoring data for similar workplaces/production facilities. In other cases, exposures may be estimated for ecological receptors such as fish by modeling releases from a facility, passage through a wastewater treatment works, and dilution in a receiving body of water. Estimation of exposures to nanomaterials are complicated by, e.g., agglomeration of nanomaterials in air, and sorption of nanomaterials to organic matter in water, and additionally, nanomaterials may also be transformed in the environment.

The European Agency for Safety and Health at Work has published guidance on handling nanomaterials at work (OSHA, 2009; 2012), as has the European Commission (2019b). NIOSH (US National Institute for Occupational Safety and Health) has issued a series of publications concerning nanomaterials at work (NIOSH et al., 2018). The OECD published a series of reports evaluating tools and models used in assessing occupational and consumer exposure (OECD, 2021a-c) and the environmental exposure to nanomaterials (OECD, 2021d).

### 4.2 Environmental exposure and quantification

The production and use of nanomaterials lead to both unintentional and intentional environmental release (e.g., Nowack et al., 2011; Giese et al., 2018). Wear and erosion from general use may lead to diffuse releases of (nano)particles. Environmental safety assessment is traditionally evaluated compartment by compartment, e.g., soil, air, water and sediment, and the fate and behavior describes the transport of the substance between different compartments. This approach is also followed for nanomaterials. To assess the exposure there is a need to determine the amount of nanomaterial released to the environment, but low nanomaterial concentration, structural heterogeneity, and the possible dynamic transformation in complex environmental matrices complicates the quantification (Lowry et al., 2012; Jiang et al., 2022). The possible transformation underscores the need to understand the fate and behavior of nanomaterials, e.g., whether they retain their nominal nanoscale size, original structure, homo- and hetero-agglomeration and -aggregation, transformation, dissolution, and corona formation, and reactivity in environmental systems. Furthermore, the natural environment has an abundance of different particles present, including nanoparticles, but only a small fraction originates from manufactured nanomaterials. Analytical techniques for identifying and measuring nanomaterials quantitatively in environmental systems were scarce early on (Nowack and Bucheli, 2007), but over the last decades the number of techniques has increased, and the measurement capability has improved (Jiang et al., 2022), but analytical tools are usually not capable of distinguishing the natural from manufactured nanomaterials (Wagner et al., 2014). There are still significant methodological gaps and, e.g., the OECD GD on Aquatic Toxicity Testing of Difficult Substances and Mixtures (OECD, 2019a) would need significant modifications to specifically consider the hazard implications related to the particulate nature of nanomaterials.

The transport between environmental compartments is important as the severity of adverse effects may depend on the compartment. In general, due to their small size individual nanoparticles are unlikely to exhibit significant settling under normal gravitational conditions in air and water. In air, nanomaterials may likely exhibit an atmospheric residence time from minutes to days (John et al., 2017). Moreover, compared to dissolved species, diffusivity in environmental media is significantly altered and thus models for environmental intermedium transport behavior of nanomaterials will likely employ and emphasize kinetic approaches. Nanoparticles in air are captured *via* filters and metallic nanoparticles can be analyzed by, e.g., single particle inductively coupled plasma mass spectrometry (Torregrosa et al., 2023), which can identify individual particle composition.

Also the sources of exposure of soil to nanomaterials and their fate in soil have been considered (Nowack and Bucheli, 2007; Cornelis et al., 2014; Hochella et al., 2019; Sun et al., 2022; Cornelis, 2025). Sources are, e.g., atmospheric deposition of nanomaterials emitted to air or water by industrial processes and by application of various consumer products. Cornelis et al. (2014) reviewed studies on fate and bioavailability of nanomaterials for natural and standard soils; bioavailability is predominantly determined by the soil’s salinity, texture, pH, concentration, and nature of mobile organic compounds and degree of saturation.

The OECD has developed TGs and GDs relevant for assessing the environmental fate and behavior of nanomaterials, see Table 1, and evaluated tools and models used for assessing environmental exposure to manufactured nanomaterials (OECD, 2021d).

### 4.3 Quantitation of exposure at the workplace

Occupational exposure is perceived to have the highest frequency, duration and level of exposure compared to other human exposure. Therefore, considerations for human exposure often focus on occupational exposure during production or use of nanomaterials. For human exposure, three main routes are usually evaluated: oral, inhalation and dermal exposure. As many commercial nanomaterials are dry powders, exposure *via* inhalation was, from the beginning, identified as a route of major importance. Inhaled (nano)particles and (nano)fibers, possibly agglomerated and covering a size range from a few nanometers to several micrometers, can deposit in the respiratory system (Kreyling et al., 2006; Braakhuis et al., 2014). The number of nanoparticles in air, including at the workplace, can be measured by condensation particle counters, whereas optical particle counters are incapable of measuring particles with diameters below 300 nm (Balendra et al., 2024). The OECD is developing a TG on Dustiness measurements for manufactured nanomaterials, which is based on CEN standards (CEN, 2019a; b; c; d; e); dustiness is defined as the propensity of a material to generate airborne dust during its handling (Liden, 2006). Given the importance of inhalation exposure, guidance on monitoring of exposure at workplaces has been published by NIOSH (NIOSH, 2022). NIOSH has also investigated the use of Raman spectroscopy for the detection of nanosized carbon compounds, as it could provide lower detection limits for them in workplace air (Zheng and Kulkarni, 2019), as compared to earlier methods used by NIOSH to detect carbon nanotubes in workplace air. Raman spectroscopy methods have been further refined by adding standardized particulate capture methods which aid in quantification of nanosized carbon compounds by Raman spectroscopy (Beobide, et al., 2024). Once inhaled, the nanoparticles will distribute in the lungs according to size and may translocate from the lungs to systemic regions; models have been developed for these phenomena (Kolanjiyil and Kleinstreuer, 2013a; b).

Early on for dermal exposure, it was unclear whether some nanomaterials would penetrate the skin, translocate, and cause adverse effects in organs, including the skin. Cutaneous absorption of different types of nanoparticles in skins from different animals and using *in vivo* and *in vitro* methodologies was reviewed by Saweres-Argüelles et al. (2023). For *in vitro* studies, this review indicated that the nanoparticles barely pass the stratum corneum. For *in vivo* studies, the review mainly focused on uptake of nanoparticles when applying sunscreens. Nanoparticles were found in the stratum corneum in all cases, but only exceptionally in blood and organs. Hence, intact skin appears to be a good barrier to prevent systemic exposure. External factors, such as UV exposure, body temperature, and the health of the exposed person or skin, can influence nanoparticles’ permeability (Marquart et al., 2020; Wang et al., 2018; Crosera et al., 2009).

The oral route is less relevant in occupational settings; however, ingestion is a potential route of exposure to nanoparticles, e.g., due to clearance from the upper airways or accidental ingestion through contaminated skin.

Basinas et al. (2018) assessed the relevance of the different routes and physical nanomaterial forms in the context of occupational exposure and protecting workers during the manufacture, handling, or end-use of nanomaterials, including an assessment of the quality of the data concerning the routes, form and likelihood of exposure for workers across the nanomaterials’ life cycle. They found evidence that all three exposure routes are of relevance for workers handling nanomaterials, and that the main route of exposure depends on how the nanomaterial is available. For dry powder nanomaterials inhalation exposure would be the main route whereas for dispersed nanomaterials the dermal route would be more relevant. Inhalation exposure may lead to dermal exposure due to direct deposition on the skin and transfer from contaminated surfaces/objects deposition and transfer resulting from the release of nanomaterials to the workplace environment. Dermal exposure may lead to ingestion exposure.

### 4.4 Modeling of exposures in the workplace and environment

For exposure, significant efforts have been directed towards modelling exposures, in addition to collecting monitoring data. Monitoring methods for nanomaterials are needed to generate the bases for models that estimate exposures; furthermore, monitoring methods specific to nanomaterials are becoming more sensitive and cost-effective. Some of the several exposure models available for general chemicals have been adapted to nanomaterial exposure. The OECD evaluated the adequacy for nanomaterials of 32 exposure models/tools for assessing occupational and consumer exposure (OECD, 2021a; b; c), most of which were deemed adequate to assess nanomaterial exposure. Among the models/tools analyzed were several models/tools recommended by ECHA (European Chemicals Agency), which were all deemed adequate for assessing nanomaterial exposure, except ECETOC TRA (European Centre for Ecotoxicology and Toxicology of Chemicals Targeted Risk Assessment) (table 61 of OECD, 2021a). In the USA, one model used to assess potential workplace exposures to industrial chemicals in general in a regulatory context under the Toxic Substances Control Act (TSCA) is The Chemical Screening Tool for Exposures and Environmental Releases (ChemSTEER) model. It can be used to estimate workplace exposures and environmental releases of chemicals manufactured and used in industrial/commercial settings (US EPA, 2025a). ChemSTEER is also used to assess nanomaterial exposures in the workplace, although it is not specifically designed to address nanomaterials.

For environmental exposure, Keller et al. (2024) noted the major scientific advances for predicting environmental concentrations (PEC) of nanomaterials, which is fundamental for exposure and subsequent risk assessment. Two main types of models are available a) material flow models and b) environmental fate models (EFM). Material Flow Analyses (MFAs) for nanomaterials are now available that take into account nanoparticle form, size distribution, dynamic release and better-informed release factors. In the environment the nanoparticles are subject to fate processes, as described above, which are accounted for to differing degrees only by EFMs, which estimate particle flows and concentrations in the environmental compartments, using input from MFAs. While the models help to understand nanoparticles in the environment, neither type has been fully validated with observed data, as field studies, which provide data sets that allow a true validation of the PECs, are still unavailable. MFAs need input data that is based on market data to estimate the production of nanomaterials, which currently has important gaps and large uncertainties. Nonetheless, a major progress is observed in the tools for generating PECs. Also, Nowack (2017) noted that a critical issue for all environmental exposure models is the missing validation of PEC values by analytical measurements, though validation on a conceptual level is possible. In the US, site-specific environmental aquatic exposures (and general population and consumer exposures) are estimated using the E-Fast screening model (US EPA, 2025b); for aquatic exposures in freshwater systems, the site-specific parameters are entered; if no detailed location data is available, generic industry codes can be applied.

Schwirn et al. (2020) analyzed challenges in environmental hazard and exposure assessment for regulatory safety assessment of nanomaterials. The challenges include accounting for exposure concentrations, including dissolving nanomaterials, in aquatic toxicity test systems and in terrestrial systems.

### 4.5 Biological tissues

The quantification of nanomaterial in biological tissues can be a challenge as the background concentration of the same elements (and/or other noise) as in the nanomaterial can be high and nanomaterial concentration can be low, necessitating extraction of the nanomaterial from the biological matrix (Monikh et al., 2019; Vladitsi et al., 2022; Laycock et al., 2022). When selecting an extraction protocol both the nanomaterials and the matrix composition needs to be carefully considered to avoid transformation of the nanomaterials or genesis of new particulates. This extraction is not straight forward and requires techniques such as digestion (e.g., enzymatic, acid, alkaline), liquid-liquid extraction, centrifugation, di-electrophoresis, and preparatory field-flow fractionation (Saleh, 2020; Jiang et al., 2022). Given the diversity of nanomaterials and matrices, no universal extraction protocol can be recommended.

Furthermore, identifying element(s) in tissue would not always be sufficient to confirm the presence of nanoparticles, as illustrated by ZnO and Ag, both of which can dissolve and could be present as Zn^2+^ and as Ag^+^. Conversely, nanoparticles may also be generated *in situ,* e.g., in plants (Lindner et al., 2022) or induced by the presence of implants in humans (Tschiche et al., 2022).

## 5 Hazard testing and assessment

Rasmussen et al. (2018) and Rasmussen et al. (2023) provide an overview of progress of developing TGs and GDs relevant for nanomaterials, see also Table 1, and information on supporting initiatives. Additional initiatives have investigated the needs and possibilities for ensuring the applicability and availability of testing methodologies for nanomaterials, for example the EU’s Horizon 2020 project ProSafe (ProSafe, n.d.). ProSafe, among others, delivered an analysis of the degree to which TGs and GDs address nanomaterials and identification of gaps, concluding on the needs and possibilities for assuring the availability of OECD TGs for testing nanomaterials (Steinhäuser and Sayre, 2017).

Furthermore, the so-called Malta Initiative, which is a voluntary network of mostly European countries (see https://malta-initiative.org/), looks for possibilities to ensure the availability of OECD test methods for nanomaterials (Heunisch et al., 2022). The Malta Initiative has connected with the EU’s Horizon 2020 NanoHarmony co-ordination action (NanoHarmony, n.d.), which developed OECD TGs and GDs using existing scientific knowledge and data. NanoHarmony coordinated the collection and use of data, organized a sustainable cooperation network between stakeholders. Some EU Horizon 2020 projects addressed the availability of methods and, e.g., RiskGONE (https://cordis.europa.eu/project/id/814425), NANORIGO (https://cordis.europa.eu/project/id/814530/en) and Gov4Nano (https://cordis.europa.eu/project/id/814401) made advances in standardizing guidance for characterizing and testing nanomaterials, supporting the adaptation and development of several OECD TGs and GDs for nanomaterials, as did the NanoHarmony (https://cordis.europa.eu/project/id/885931/results) coordination action. These projects have also provided an overview of the need for developing additional OECD methods for nanomaterials (Bleeker et al., 2023; Rasmussen et al., 2023).

The EU publishes regulatory test methods in the Test Methods Regulation, which is regularly updated (European Council, 2008) and most often these are methods endorsed in the OECD TGP, though also EU specific methods can be included.

Toxicological assessment is fundamental for ensuring the safe use of nanomaterials, and the availability of *in silico*, *in vitro* and *in vivo* methods is fundamental. However, most of the existing approaches for *in silico*, *in vitro* and *in vivo* methods were and are developed for conventional chemicals and require additional considerations, validation, and adaptations when applied to nanomaterials.

### 5.1 *In vivo* testing protocols for hazard assessment and biodistribution

For safety assessment of chemicals, data on their possible hazardous properties is fundamental, to be related to the measured or estimated exposure. Possible nanomaterial hazards may be affected by physico-chemical characteristics such as particle shape, size and surface area, which can affect both the mode of action and toxicokinetics of particles (Shin et al., 2015).

Additional OECD TGs or GDs for *in vivo* testing may be needed in the near future. For example, while recent progress has been made on environmental testing protocols, particularly with regard to aquatic ecotoxicity (OECD (2021f)), more guidance on aquatic ecotoxicity testing is needed. Thus, Pulido-Reyes et al. (2024) has recommended specific flow diagrams to determine the method to be used to test for toxicity of nanomaterials using OECD TG 203 (OECD, 2019b). These flow diagrams suggest how pre-testing should specifically be conducted for nanomaterials with varying solubility/dissolution, and agglomeration potential. They might be relevant for and adaptable to other types of testing, which would require additional work. Further, specific methods for dispersion of nanomaterials in stock solutions, dispersion additives, conducting pre-tests with various nanomaterials, and maintaining concentrations in test solutions are recommended. Pulido-Reyes et al. (2024) also note that such approaches applicable to fish acute tests could be applied to fish chronic tests, although specific recommendations for other durations are not provided; neither are modifications provided for other test species, such as sediment dwellers.

### 5.2 Alternative methods

#### 5.2.1 Challenges for nanomaterials

The importance of availability of alternative methods is increasing as fulfillment of regulatory toxicological information requirements moves towards using *in vivo* data as a last option (e.g., European Parliament and Council, 2006). Furthermore, the shift towards human relevant models has pushed for the development of *in vitro* and *in silico* alternative methods, also called New Approach Methodologies (NAMs), and especially human-based test systems, which aim at the 3 Rs (Reducing, Refining and Replacing animal models). Alternative methods can be *in silico* methods, or *in vitro* methods, i.e., use tissues, reconstructed tissues, whole cells or parts of cells, or employ a reduced number of animals.

As stated, relevant, reliable and validated TGs should be developed in accordance with OECD GD 34 (OECD, 2005), which is a lengthy process. Classically, toxicology relies on *in vivo* methods, making the demonstration of regulatory relevance of alternative tests cumbersome, as one-to-one replacement of *in vivo* tests with alternative tests is rarely possible. Regulatory toxicology assessment is in general moving towards applying *in vivo* testing as a last resort, which has accelerated the development of alternative approaches, which include *in vitro* tests, grouping approaches and other NAMs. The issues around nanomaterials, e.g., that toxicity may depend on particle size and the possible high number of toxicological tests needed to cover different nanomaterials of the same composition, have increased the interest in testing nanomaterials by alternative methods. The development and use of alternative methods, including NAMs, is overseen by the International Cooperation on Alternative Test Methods (ICATM), the OECD, and other (national) bodies. However, significant time is needed for alternative methods to arrive at an OECD TG.

Currently, there are only few OECD TGs with alternative methods for testing nanomaterials. Several high priority *in vitro* TGs for key endpoints are not applicable to nanomaterials (Doak et al., 2012), including the bacterial reverse mutation test (OECD, 2020e). Some, however, are relevant to nanomaterials as written, such as the OECD TG 439 (OECD, 2021h). The OECD has published two GDs relevant for alternative tests (OECD, 2022b; 2023a). Table 1 lists *in vitro* TGs and GDs that have been or are being adjusted to nanomaterials: a GD for the adaptation of *in vitro* mammalian cell-based genotoxicity TGs for nanomaterials; an *in vitro* comet assay for testing genotoxicity of nanomaterials; and two tiered-testing or IATA efforts which may use *in vitro* tests to assess bioaccumulation in ecological organisms, and separately to assess the intestinal fate of ingested nanomaterials in mammalian species. The current focus is thus appropriately on achieving nanomaterial-relevant OECD genotoxicity protocols, given the importance of genotoxicity endpoints for regulatory review, and the potential for nanomaterials to interact cellular components that could affect genotoxicity. Doak et al. (2023) reviewed the current state of genotoxicity protocols for nanomaterials, as applied to regulatory submissions.

Additional TGs for alternative testing of nanomaterials are underway, beyond the development of IATAs and *in vitro* protocols. A nanomaterial-relevant AOP is being developed for prediction of pulmonary fibrosis (Halappanavar et al., 2023) and it has been specifically used by researchers to develop both *in vitro* and *in silico* methods for screening and prioritizing the ability of nanomaterials to cause adverse pulmonary effects. It is currently qualitative, and though there is some evidence of dose-response relationships, they are unavailable for each individual key event in this AOP.

Thus, NAMs, including not-yet-validated methods, are being explored as a next approach to screen materials and obtain indicators on the need for and nature of further testing (Cattaneo et al., 2023; Haase et al., 2024; Sewell et al., 2024; van der Zelm et al., 2022; ECHA, 2024b).

For whole cells, test systems based on two- (i.e., a monolayer cell culture, or cells dispersed in a liquid) and three-dimensional cell cultures are emerging (Urzi et al., 2023). Additionally, knowledge frameworks are employed to integrate information, all of which are promoted by the OECD. One is Adverse Outcome Pathways (AOPs) that support chemical risk assessment based on mechanistic reasoning (OECD AOP, 2025a.; Halappanavar et al., 2020) also *via* an AOP-Wiki. An AOP is a model that identifies the sequence of molecular and cellular events leading to a toxic effect after exposing an organism to a substance (https://ntp.niehs.nih.gov/whatwestudy/niceatm/comptox/ct-aop/aop). AOPs have been proposed by OECD as a way to develop IATA, which are flexible approaches for chemical safety assessment based on the integration and translation of the data derived from multiple methods and sources (OECD, 2017a). Furthermore, “omics” technologies (e.g., transcriptomics, proteomics and metabolomics (Dai and Shen, 2022)) are proposed, which allow identifying potential toxicity pathways, which may lead to insights into adverse health effects.

For nanomaterials, a further complication is that during testing particulate nanomaterials may interact with the cells differently from soluble materials, by sedimenting on the cells or floating to the surface, giving raise to artefacts (Guadagnini et al., 2013). Artefacts can be overcome by following standardized protocols that ensure meaningful and reproducible quantification of the *in vitro* dose, with consistent measurement and reporting between laboratories, and standardized and integrated methodologies for the generation of stable nanomaterial dispersions in cell culture media. The dispersed nanoparticles should be characterized, especially size distribution and effective density (ISO, 2024), which are the main properties that determine particle kinetics in an *in vitro* system. Finally, the protocols should describe the determination of the nanomaterial dose delivered to cells over the course of the *in vitro* exposure based on robust numerical fate and transport modeling (DeLoid et al., 2017).

#### 5.2.2 *In vitro* test systems

Many current *in vitro* systems are two-dimensional and lack interactions with other cells and connective tissues. Air-liquid interface (ALI) co-cultures are being developed which mimic more closely pulmonary *in vivo* conditions and dosing. Longer duration, more realistic *in vitro* systems are now also being combined with other alternative methods such as AOPs and *in silico* predictive algorithms. For example, Barosova et al. (2020) utilized an *in vitro* system, addressing many of the points raised by Drassler et al. (2017), to test two different types of carbon nanotubes (CNTs) in an organotypic three-dimensional model that mimics human alveolar tissue at the pulmonary air-blood barrier. This ALI system conforms to some of the key events in the OECD AOP for pulmonary fibrosis: hence, this *in vitro* system follows some of the mechanisms assessed by regulators that lead to fibrosis. Beyond more realistic *in vitro* models, numerous systems are being developed for pharmaceutical applications, which may have future applicability to nanomaterial hazard assessment. Systems such as organoids and 3D bioprinting of cell based functional structures could offer further similarities to *in vivo* toxicity responses, beyond those such as the ALI system. Organoids are comprised of organ-specific cells and self-organize into organ-like three dimensional tissue systems which have advanced features for testing nanomaterials (Shen et al., 2023). Advantages of organoids for nanomaterial toxicity assessments include the following ways organoids mimic the tissue micro-environment (Nabi et al., 2022): numerous cell types mimic tissue architecture; the actual flow of nanoparticles in tissues is simulated; cellular functions like migration, differentiation, and apoptosis occur; and some systems allow longer observation periods such that cumulative effects can be seen. Organoids can be improved through 3D bioprinting, which allows more reproducible organoids to be produced that also have features such as improved vascular systems and larger sizes (He et al., 2023).

#### 5.2.3 Biodurability testing

Another area under development by the OECD has been biodurability testing as a broad indicator of persistence and possible adverse effects (OECD, 2018d). Both *in vitro* and acellular assays were examined as they apply to biopersistance of nanomaterials in lung, gastric and intestinal fluids. Given the interest in biopersistance as an indicator of long-term health effects due to pulmonary exposure, the use of artificial lung lining fluids and artificial lysosomal fluids to test biodurability remains very pertinent. Such tests would serve as preliminary testing to the OECD *in vivo* acute and subchronic inhalation tests for nanomaterials (OECD, 2018b; c), and could complement the OECD AOP efforts noted above. The OECD recently associated the effects of lysosomal dysfunction in connection with the pulmonary fibrosis AOP, further reinforcing the utility of assays that show persistence in lysosomal fluids (OECD, 2020a). Acellular assay results could divide inhaled nanomaterials into those which are less biopersistent (and possibly cause short-term effects) and those which are more biopersistent (leading to long-term adverse effects). Interest has been expressed in these acellular assays for nanomaterials by at least one additional regulatory body: NIOSH has examined beryllium dissolution rates in lysosomal fluids as an indicator of the potential of beryllium compounds to cause chronic beryllium disease following inhalation exposures (Stefaniak et al., 2006). Such groupings could be key to regulatory submissions for groupings and/or screening approaches for panels of nanomaterials (as part of IATA). Murphy et al. (2021) proposed both lysosomal and lung fluid biopersistance assays as part of the early tiers of an IATA that identifies nanomaterials with potential to cause mesothelioma. EFSA (European Food Safety Authority) has recognized the utility of dissolution tests in gastrointestinal and lysosomal fluids as part of a rationale to waive certain *in vivo* studies for assessment of food and feed risks (More et al., 2021a).

#### 5.2.4 Development of New Approach Methodologies

The EFSA project NAMS4NANO, ‘Integration of NAMs results in chemical risk assessments: Case studies addressing nanoscale considerations’, reviewed legal requirements and possibilities for change (Cattaneo et al., 2023; Vincentini et al., 2023). Through the use of NAMs, EFSA aims at a Next-Generation Risk Assessment, integrating NAMs data in risk assessment, as promising tools in food-related nanomaterial safety assessment, which includes (a) nanomaterials as defined in EU food legislation (European Parliament and Council, 2015), (b) non-nanomaterials containing a fraction of nanoparticles, and (c) nanostructured materials with nano-scale characteristics. In case (a), the material is intentionally engineered at the nanoscale to achieve specific properties, and in general, toxicological information on the non-nanomaterial(s) is available. To cover nanospecific considerations based on mechanistic understanding of toxicokinetic and toxicodynamic processes at the nanoscale, the EFSA Guidance (More et al., 2021a) suggests the use of read-across and the development of NAM-based IATA. In case (b), the main issue is that existing studies have not *a priori* included nanospecific considerations. To avoid conducting new *in vivo* studies, EFSA guidance (More et al., 2021b) suggests using NAMs to fill the data gaps and complement the available studies.

Furthermore, experts will evaluate the potential of using NAMs in EFSA’s risk assessments of chemicals with the goal to develop a qualification system for NAMs and internationally harmonized guidance on the use of NAMs in EFSA’s risk assessments. The EFSA approach is important for all EU legislation addressing nanomaterials as the EU works towards a “One Substance–One Assessment” approach.

In the USA, the use of new NAMs for regulation is overseen by ICCVAM (US Interagency Coordinating Committee on the Validation of Alternative Methods). Currently, 17 US Federal Agencies are involved in this process. A Strategic Roadmap for the development of NAMs was published in 2018 (ICCVAM, 2018), which notes that ICCVAM efforts at validation of NAMs need to still rely on the principles of OECD GD 34 (OECD, 2005) to obtain acceptance by regulatory authorities. However, ICCVAM sees a need to develop new approaches for achieving validation of NAMs to allow incorporation of 21st-century science into modern risk assessment and hazard identification in a timely manner: the OECD GD 34 does not fully address all considerations required for the effective evaluation of many modern technologies and approaches. It allows a great deal of flexibility *via* a “modular approach” to validation, which was, however, not usually applied to ICCVAM-coordinated validation studies and hence greatly augmenting the expense and duration of these studies. Thus, the overarching principles described in GD 34 need to be incorporated in a more flexible and efficient manner. Of particular interest is also the concern of Industry related to the lack of clear guidance on the acceptability of NAMs. The US EPA has recently listed all NAMs which can be used for the assessment of new industrial chemicals and pesticides under regulatory review (US EPA, 2021); some of these NAMs are applicable to the testing of nanomaterials.

While new partnerships and more effective use of data are suggested, specific standards and guidance on how to validate NAMs for regulatory use are not yet available. Considerations such as *in vivo* validation of *in vitro* effects methods across a representative range of nanomaterials raise concerns regarding the resources and time needed for traditional validation approaches. Some publications, such as Petersen et al. (2021), indicate that there are considerations for *in vitro* tests that should be addressed for use of NAMs for any regulatory purpose in the U.S. such as screening, prioritization, or replacement of *in vivo* tests. These include careful evaluation of dosimetry, and the use of interference/bias controls for nanomaterials which GD 34 does not identify in detail sufficient for nanomaterial testing.

The future of NAM development for predicting the hazard of nanomaterials could proceed *via* two different paths. The first could focus on simpler methods, not necessarily validated for regulatory acceptance, generally intended for use by industry as part of screening processes in a “Safe by Design” approach (Ruijter et al., 2023) and in the European Commission’s framework for “Safe and Sustainable by Design” (Caldeira et al., 2022; Abbate et al., 2024; Garmendia et al., 2025). The second path would focus on alternative methods that are acceptable to regulators to use directly to predict a hazard endpoint. Such methods should be validated (typically *via* the OECD) to be acceptable in a regulatory review that precedes commercialization of a nanomaterial (Drassler et al., 2017). Only few such *in vitro* methods have cleared the OECD validation process. EFSA is looking into a new approach to the use of NAMs: “qualified”, not fully validated NAMs that are accepted for very specific regulatory purposes, see above. Drassler et al. (2017) provided a summary of the criteria to consider for validation of a new *in vitro* method.

For environmental assessment of chemicals, *in vitro* methods may be relevant (Rehberger et al., 2018), based on research into the use of *in vitro* cytotoxicity assays with fish cells as non-animal alternative to the *in vivo* lethality test with fish and on *in vitro* biotransformation assays as part of an alternative testing strategy for bioaccumulation testing with fish. This has, among other led to the OECD scoping review “A Tiered Approach for Reliable Bioaccumulation Assessment of Manufactured Nanomaterials in the Environment Whilst Minimising the Use of Vertebrate Testing” (OECD, 2024b).

#### 5.2.5 Grouping of nanomaterials

Grouping and read-across approaches, based on similarity between substances, are well-accepted alternative methods to toxicity testing in regulatory submissions for general chemicals (ECHA, 2024a; US EPA, 2024b; b; OECD, 2017b). Hence, the fate, toxicokinetics and/or ecotoxicity of structurally similar “target” chemical(s) are predicted based on available data on “source” chemicals. These methods rely on similarities of grouped chemicals such as physico-chemical characteristics, persistence, and/or toxicity/mode-of-action; they also can rely on *in vitro* test results to support conclusions for less-tested chemicals in the grouping. For nanomaterials grouping and read-across is of particular interest as small changes to the nanomaterial may result in different nanomaterial properties, and characterization of the nanomaterials would be an important element for grouping and followed by read-across, and links between physico-chemical properties and ecotoxicological effects are also explored (OECD, 2016b). However, several challenges remain, including the identification of the most relevant physico-chemical properties to support a claim of nanomaterial similarity, noting also that in general, the proper physico-chemical description of a nanomaterial requires additional parameters, which, *inter alia*, may change during the life cycle a NM, indicating that also the ecotoxicological profile may change.

Grouping approaches have been specifically suggested by the US EPA for carbon nanotubes for several endpoints (including physico-chemical characterization, pulmonary toxicity, and worker exposure) and uses; these approaches are compatible with the way in which the US EPA assesses the risks of chemicals in general (Godwin et al., 2015). These methods are part of a broader methodology to testing and assessment that utilizes all relevant and reliable existing information to assess chemicals in a regulatory context: IATAs. There is one OECD IATA case study that has been developed for nanomaterials (OECD, 2018a). IATAs would be at least as useful for filling data gaps for nanomaterials as they are for traditional chemicals: there are many physico-chemical variants of any given nanomaterial class such as carbon nanotubes, and limited existing effects data for such variants that are acceptable to regulators. ECHA has proposed grouping approaches specifically for read-across of nanoform hazard data. “A set of similar nanoforms” can be created, when it is possible to conclude that the hazard assessment, exposure assessment and risk assessment of these nanoforms can be performed jointly for all endpoints (ECHA, 2019b). EFSA has also issued guidance on the grouping of food and feed nanoforms for read-across, which refers to the ECHA guidance for assessment (More et al., 2021b). In addition, EFSA also suggests examining a current European research-generated read-across framework for nanomaterials which has specific examples of nanomaterial read-across hypotheses and detailed guidance to construct groupings and IATAs that may be acceptable to regulators (GRACIOUS, 2021). The GRACIOUS framework was developed based on ECHA guidance, and offers a number of pre-defined hypotheses and related IATAs which are based nanomaterial regulatory guidance (Stone et al., 2020; Murphy et al., 2023).

The OECD has published guidance on grouping (OECD, 2017b), which also reflects outcomes of two workshops on categorization and grouping for nanomaterials (OECD, 2016a; 2016b), however no specific guidance could be given for nanomaterials. Subsequently, e.g., EU projects (Guisti et al., 2019; Stone et al., 2020) created a sufficient knowledge base. Thus, the ongoing update (2025) of the OECD guidance on grouping includes guidance for grouping of nanomaterials. Stone et al. (2020) provides an initial set of hypotheses for the grouping of nanoforms which take into account both the purpose of grouping and the identity and use(s). An appropriate pre-defined grouping hypothesis can be selected based on an initial collection of basic information, which also allows selection of a tailored IATA, designed to generate new evidence to support acceptance or rejection of the hypothesis. Groupings include those for inhalation effects, gastrointestinal effects, and dermal effects. The framework also supports users who want to develop their own user-defined hypothesis (and IATA). In addition, the IATA guides acquisition of the information needed to support read-across.

Occupational nanomaterial inhalation exposures are frequently a concern, and there is a shortage of OECD-compliant subchronic and chronic rodent inhalation studies for many specific nanomaterials. As a result, grouping approaches have been proposed by various organizations to estimate acceptable occupational exposure levels for workers. Examples of methods which seek to establish control bands for setting worker exposure limits *via* grouping of nanomaterials have been developed by US and European organizations (Liguori et al., 2016). Some of these control banding approaches accept data from studies other than those OECD-compliant methods just noted. Control banding methods have been reviewed by NIOSH (Dunn et al., 2018). NIOSH tested six current control banding methods against six different nanomaterials (four of which had OELs (occupational exposure levels) generated by non-regulatory bodies). The conclusion was that while the control banding methods appeared to be conservative and protective, more data are needed in order to validate such control banding methods. For example, the workplace exposure measurements, workplace exposures as compared to dustiness measurements, inhalation toxicity data need additional data for method validation. Additional OELs accepted by regulators are needed to compare with outcomes of the control banding tools. Given the shortage of regulatory OELs for nanomaterials, and the diversity of nanomaterials coming into commerce, NIOSH has since provided a framework to assess various control banding tools, as more data to validate these approaches come forward (NIOSH, 2021). The opinion from NIOSH can allow US regulatory agencies to set regulatory exposure limits for workers in the United States.

## 6 Risk characterization

The OECD Council Recommendation (OECD, 2013 and 2017) notes that methodologies, including risk assessment (OECD, 2022a), that are applied to general chemicals can, when appropriately modified, be used for nanotechnology as well. Regarding risk characterization, it is important that the hazard and exposure data are directly comparable. This means not only that the hazard and exposure data must have the same unit, they must also concern the same nanoform, or at least sufficiently similar nanoforms. For nanomaterials this remains a major challenge.

Box 1 provides an illustrative case study of risk characterization of carbon nanotubes.

Box 1 **Example: A case study from the USA of Carbon Nanotubes (CNTs), illustrating a new technology and issues related to data availability: a shortage of available pulmonary effects and ecotoxicological tests on CNTs whose data are acceptable to regulators (per OECD guidance and guidelines) results in difficult and potentially overly conservative regulatory decisions.**

**Background:** Certain classes of nanomaterials are currently commercialized and incorporated into many industrial and consumer products. However, the amount of human health and ecotoxicity data acceptable to regulators lags far behind, and there is a need for more data to support certain groupings such that regulators can apply read across or interpolation approaches to determine the potential effects of a new nanomaterial. This example presents some of the issues encountered for CNTs, and almost the exact same situation exists for other highly-relevant nanocarbons such as graphene-based materials and carbon nanofibers.

An **example** of such a group of nanomaterials is CNTs. They conform to the fiber paradigm which links CNTs to potential adverse effects *via* the pulmonary route of exposure. Only one CNT has been identified as being carcinogenic in a full rodent *in vivo* pulmonary assay that is compliant with OECD guidelines: the Mitsui-7 multiwalled CNT (MWCNT) was identified as carcinogenic by Kasai, et al. (2016). IARC (2017) concluded that this same Mitsui-7 MWCNT is possibly carcinogenic to humans, *via* the pulmonary route of exposure, even prior to their review of Kasai, et al. (2016). No other CNTs were identified as possibly carcinogenic to humans by IARC. There is, however, a critical need for additional subchronic to chronic pulmonary effects testing of CNTs with various physico-chemical properties to better understand the range of effects possible, as key physico-chemical properties are varied. Below is illustrated some of issues concerning the use of CNTs in batteries, and regulatory decisions resulting from the limited availability of both mammalian pulmonary toxicity data and ecological effects data which meet OECD standards for acceptability. The lack of such data for both mammalian and ecological effects leads to an inability to form valid groupings of carbon nanotubes for these endpoints to enable analogue selection and/or interpolation. Furthermore, in the case of ecological effects, it has led to a conclusion that no aquatic or terrestrial ecotoxicity value can be assigned to carbon nanotubes.

**Issues for data availability**: **CNT and carcinogenicity studies.** Carbon nanotubes and various graphene-based nanomaterials can enhance the performance of lithium-ion batteries for electric vehicles (Yuan et al., 2016). Given the environmental advantages of electric vehicles (EV) vis-a-vis air pollution and global warming tailpipe emissions, the EV market will likely continue to grow in the coming years. The battery technology has involved the use of CNTs (Lopez, 2022), and to be profitable the manufacturing plants operate at very large scales. Some of the processes involved in the manufacturing of these batteries involve cutting anode or cathode sheets coated with CNTs, and other processes which can generate dust in the workplace (Liu, et al., 2021). The unavailability of appropriate inhalation toxicity test results for CNTs have led to regulatory barriers to production of vehicle batteries (Fadeel and Sayre, 2023). These conservative regulatory decisions, and resulting costly controls for very large EV battery manufacturing plants, are driven in part by the toxicological uncertainty of the CNTs used.

Regulatory agencies often require at least subchronic inhalation toxicity data on a CNT, or subchronic inhalation toxicity data on a comparable analogue CNT, to determine the potential toxicity and carcinogenicity potential of a new CNT that is to be used commercially in applications such as the manufacture of EV batteries. The applicable test guideline for subchronic inhalation toxicity assessments is the OECD 413 TG (OECD, 2018C). All available subchronic inhalation studies that meet OECD standards for acceptability were gathered to assess the pulmonary effects of carbon nanotubes and nanofibers in 2013 (NIOSH, 2013). NIOSH noted only two subchronic inhalation studies on which to base a “Recommended Exposure Limit” for workers: a 90-day study by Pauluhn (2010) on an underivitized MWCNT referred to as Baytubes^2^ and a second study on an underivitized few-walled MWCNT by Ma-Hock, et al. (2009) referred to as Nanocyl NC 7000 (a few-walled MWCNT, 0.1–10 µm long, 5–15 nm wide, BET surface area 250–300 m^2^/g, agglomerated, 9.6% aluminum oxide with traces of Co and Fe). No additional *in vivo* subchronic or chronic carcinogenicity studies were available. Since the publication of NIOSH (2013) only two other studies are available: a subchronic inhalation study by Kasai, et al. (2015) on an underivitized many-walled MWCNT referred to as Mitsui-7 or MWCNT-7^3^, and a follow-on carcinogenicity study on the same Mitsui-7 MWCNT by Kasai, et al. (2016). To our knowledge, there are no additional published OECD-compliant subchronic or chronic inhalation studies for any other CNTs that are in the public domain.

The small number of studies noted above (four) on only three underivatized CNTs should be considered in light of the physico-chemical properties often associated with the degree of adverse pulmonary effects seen with CNTs. These toxicity determinants include the following: purity, diameter, length, surface charge, biopersistence, solubility, degree of chemical functionalization, structural defects, and state of aggregation (Bergamaschi, et al., 2021). The International Agency on Cancer Research (IARC, 2017), a respected international body whose findings are generally accepted by regulatory authorities, has also stated that the pulmonary effects of CNTs can be directly correlated with their physico-chemical properties.

Considering the vast variation in the physico-chemical properties in underivatized CNTs alone, four studies on three different underivitized CNTs is insufficient to understand the full range of mammalian pulmonary effects due to inhalation exposure to carbon nanotubes. These four studies cannot be used alone to construct groupings of carbon nanotubes that would include the vast array of even underivatized commercial CNTs. IARC (2017) reinforces this view in its findings, which were based in part on the same two subchronic inhalation studies used by NIOSH in its 2013 publication, and on the newer Kasai subchronic study (Kasai, et al., 2015). IARC found that “MWCNT-7 multiwalled carbon nanotubes are *possibly carcinogenic to humans* (Group 2B)… Multiwalled carbon nanotubes other than MWCNT-7 are *not classifiable* as to their carcinogenicity to humans (Group 3)… and…. Single-walled carbon nanotubes are *not classifiable* as to their carcinogenicity to humans (Group 3).”

Regulatory agencies often apply approaches such as uncertainty factors and other statistical methods to arrive at conservative estimates for the potential adverse effects of a new untested CNT under review, based on available validated subchronic or chronic mammalian pulmonary study data sets generated using carbon nanotube analogues which are similar to the untested CNT under review in terms of their physico-chemical properties. These estimates of adverse effects may then be combined with similarly conservative estimates of worker exposure that results in even more conservative risk estimates, which result in restrictive engineering controls and/or comprehensive protective respiratory protection requirements.

Using this approach, EPA allowed the use of some new MWCNTs for EV battery production (US EPA, 2022). However, based on carcinogenicity concerns, the Agency required that all manufacturing processes to produce the EV batteries be conducted in “enclosed processes” which allow no releases of MWCNTs to workplace air. This prevents the use of MWCNTs in almost any battery manufacturing process that generates a dust, aerosol, or mist. Many of the standard processes and operations for manufacturing lithium-ion batteries would thus have to be fully enclosed, significantly increasing the cost of associated engineering controls. The Agency also imposed strict standards for personal protective equipment to prevent inhalation of CNTs in workplace air. EPA defines an excess cancer risk as acceptable if it is less than one additional cancer case in one million people (US EPA, 2005a). The restrictions on inhalation exposures in this case were driven by an excess cancer risk of 2.0–6.8 cases in one million people for the new CNTs regulated by EPA, and the Agency noted that these excess cancer risk estimates are based on use of Mitsui-7 MWCNT (MWCNT-7) as an analogue for the new CNT under examination (US EPA, 2022). It is unclear, due to industry’s confidentiality concerns, how similar the physico-chemical properties of the new approved MWCNTs are to the MWCNT-7 used in the EPA risk assessment. However, it is highly unlikely that these CNTs were MWCNT-7, as the company who submitted the application to EPA manufactures its own CNTs. MWCNT-7 are the only MWCNTs tested in OECD-compliant studies that have such unique physico-chemical properties; they are short (1 μm–19 µm) rigid CNTs (diameter 70 nm–170 nm) and occur primarily as singlets (IARC, 2017). These physico-chemical parameters cannot be compared to the approved MWCNTs, as FAIR information on this MWCNT’s physico-chemical properties is unavailable (due to Company confidentiality concerns). The MWCNT-7 physico-chemical properties are also markedly different from those noted above that were tested in subchronic inhalation studies, and accepted in a regulatory context by NIOSH and IARC. MWCNT-7 are the most potent of all CNTs tested to date in subchronic inhalation studies (Kasai et al., 2015; Kobayashi et al., 2017; Oberdörster et al., 2015). In any case, the data from the only chronic toxicity study on CNTs should only be narrowly applied, per IARC. Further, the few additional subchronic studies available (four, on three underivatized CNTs) encumber the ability of regulators to assess directly the potential adverse effects of carbon nanotubes in general. Hence, numerous adjustments are necessary to estimate the potential adverse pulmonary effects of a new CNT intended for commercialization. An examination of the EPA ChemView portal for additional regulatory decisions on carbon nanotubes will show that in almost every case, the new CNTs are also subject to a “no releases to air” restriction for the workplace. In some cases, other analogue CNTs are cited as the basis for pulmonary worker risks assessed; in other cases the analog CNTs are not described. Of course, companies are always encouraged to suggest alternative analogue CNTs to the US EPA to use for assessing pulmonary toxicity, but the available choices that are accompanied by acceptable pulmonary toxicity testing data are very constricted at this time. Companies are also encouraged to conduct new pulmonary toxicity testing, but given the costs of an OECD-compliant subchronic pulmonary toxicity study, this is in many cases seen as unaffordable.

**Issues concerning availability of environmental toxicity data**. As is the case with restrictions due in part to insufficient pulmonary toxicity data on CNTs, the same is true for disposal of CNTs not incorporated into batteries due in part to the lack of ecotoxicity information for CNTs (US EPA, 2022). In general, almost all regulatory decisions on carbon nanotubes examined using the US EPA ChemView portal cite a “no releases to waters of the United States” provision. This is due to the lack of valid ecotoxicity data applicable to these CNTs, as stated in the regulatory decision documents posted on the EPA portal (see applicable CNT “Consent Orders’ and their supporting risk assessment documents). The EPA relies on OECD-compliant testing for at least three freshwater aquatic organisms (fish, aquatic invertebrates, and algae) to assess a new chemical’s potential hazards. Recent reviews of the literature in both Google Scholar and PubMed also indicate that these key OECD-compliant studies are still unavailable for any single CNT. The “no releases to water” provision can trigger costly disposal methods for the liquid (and solid) waste generated in battery manufacturing: costly engineering approaches must be used to prevent releases, or wastes must be concentrated prior to disposal by incineration or landfilling. Moreover, the absence of studies (i.e., knowledge is unavailable) also means that there are very limited to no releases allowed to the terrestrial environment: hence the restrictions on disposal of solid wastes that contain CNTs.

## 7 Data quality and FAIR data

In addition to ensuring that appropriate methods for testing nanomaterials are available, the future use of generated data needs to be considered. A study by Comandella et al. (2020) evaluated the quality of physico-chemical datasets for nanomaterials stored in the eNanoMapper database (Jeliazkova et al., 2015), developed as a repository containing the curated data of research projects, to assess data completeness and variability. They found that often the physico-chemical data was incomplete, missing, e.g., information on sample preparation and standard operating procedures or uncertainty, significantly reducing the possibilities for comparing test results or re-using the data. Some of the reported main challenges for nanomaterials include the lack of persistent identifiers and of standardized user-friendly data retrieval services and reporting formats; the latter leads to a lack of crucial metadata. Furthermore, the data gaps and uncertainty over data quality may result in the data being poorly suited to, e.g., modelling requirements. Moreover, an agreed data management structure for data generated in research projects is lacking, which hampers re-use of that existing data (Jeliazkova et al., 2021). For data generated within research projects metadata are even more relevant, as, e.g., non-standardized methods are applied and thus, a detailed description of the experimental procedure is needed.

Considerations such as those outlined above led to the recognition of the importance of making the data FAIR. To achieve useful FAIR data, there is also a need to assess the data quality, curate, interpret and integrate data and metadata, noting also that FAIR data are not necessarily available for free. One way to improve the completeness of data is to develop templates that prompt for information, as well as help to structure databases for the data. Thus, the OECD TGs are accompanied by internationally recognized OECD harmonized templates (OHTs) for data collection, and OHTs 101 to 113 for physico-chemical data for nanomaterials have recently been developed (Rasmussen et al., 2019). This should ensure that all relevant metadata for regulatory testing is collected, which is of key importance for ensuring that the data is FAIR (Jeliazkova et al., 2021).

Thus, while a wealth of data has been generated for nanomaterials, the availability of FAIR data (Comandella et al., 2020; Jeliazkova et al., 2021), which is important also for grouping and read-across, is scarce, and it is a major challenge also in view of the complex and multidisciplinary nature of safety data for nanomaterials.

## 8 Reference materials and the Joint Research Centre’s (JRC) nanomaterials repository

One gap for developing methods for measuring nanomaterials became evident early on: the absence of benchmark materials for testing nanomaterials. Benchmark materials include (certified) reference materials as defined by ISO (ISO, 2018), and “representative test materials” (Roebben et al., 2013; ISO, 2018), see Box 2. Such materials must be sufficiently homogeneous and stable, so that results obtained by testing different sub-samples and at different places and different times, can be meaningfully compared. For specific purposes, these materials also need to have reliable, assigned property value(s). The importance of such materials is illustrated by the creation of a dedicated ISO Technical Committee for Reference Materials (ISO/TC 334).

Box 2 **Definitions of the terms (certified) reference material and term representative test material**

A **reference material** is a *material, sufficiently homogeneous and stable with respect to one or more specified properties, which has been established to be fit for its intended use in a measurement process* and a **certified reference material** is a *reference material (RM) characterized by a metrologically valid procedure for one or more specified properties, accompanied by an RM certificate that provides the value of the specified property, its associated uncertainty, and a statement of metrological traceability* (ISO, 2015).

*A* ***representative test material (RTM)*** *: material, which is sufficiently homogenous and stable with respect to one or more specified properties, and is implicitly assumed to be fit for its intended use in the development of measurement and test methods that target properties other than those for which homogeneity and stability have been demonstrated*

Note 1 to entry: An RTM may be a reference material for other properties (i.e., properties for which homogeneity and stability have been demonstrated), and a candidate reference material for the target property.

*Note 2 to entry: An RTM can be a useful tool in inter- or intra-laboratory developments of test methods for which reference materials cannot (yet) be produced.* (ISO, 2018).

With increasing availability of test results on nanomaterials, it became evident that the test material itself can be an additional factor of uncertainty when performing the same test of nanomaterials of the same chemical formula in different laboratories. This is because a nanomaterial is characterized by several additional properties, such as its particle size and particle size distribution, and minor modifications of the physico-chemical characteristics may lead to significant changes of, e.g., the nanomaterial’s functional or ecotoxicological properties. The availability of nanomaterials for testing from single batches would facilitate the comparability of results obtained in different laboratories and across research projects. The lack of both reference and control samples, and of harmonization of the procedures used to generate the data, are main issues preventing the full use of studies concerning environmental, health, and safety issues for nanomaterials (Krug, 2014). Over time several reference nanomaterials have become available, e.g., from the JRC, BAM (German Federal Institute for Materials Research and Testing) and NIST (USA National Institute of Standards and Technology).

To address this comparability gap, the European Commission’s JRC established the JRC Nanomaterials Repository in 2009 (Totaro et al., 2016). Its initial aim was to support the OECD WPMN by providing subsampled nanomaterials originating from the same batch, thus eliminating one source of uncertainty in the testing and promoting better reproducibility and reliability in safety testing of nanomaterials. The materials from the JRC are representative test materials (Roebben et al., 2013). Later also EU-funded research projects as well as global research and regulatory partners have used these materials as benchmarks, which has resulted in the generation of a significant amount of good quality data on these nanomaterials. These nanomaterials have become very well characterized with regard to physico-chemical properties and have been tested for ecotoxicological effects and fate, too, as part of the many tasks undertaken in research projects, e.g., MARINA (Managing Risks of Nanoparticles, https://cordis.europa.eu/project/id/263215/reporting), NANoREG (A common European approach to the regulatory testing of nanomaterials, https://cordis.europa.eu/project/id/310584) and GRACIOUS.

## 9 Discussion, conclusions and outlook

The progress of understanding nanotechnology in the context of safety, and presented in this paper, is summarized below.

### 9.1 General understanding and definition

Ca. 25 years ago, nanotechnology emerged as a key enabling technology associated with nanomaterials. The small size of nanoparticles soon raised safety concerns globally, sparking research, and discussion within the OECD, to understand whether regulatory assessment of nanomaterial safety could follow guidelines for general chemicals. A first issue was to define what ‘nanomaterial’ is in a regulatory context, leading to a converging global agreement that the nanoscale ranges from 1 nm to 100 nm. Nanomaterials have since been explicitly addressed by legislation; however, the exact definition varies with geographical region and the legislation, e.g., in the way agglomerates and aggregates are considered (Rasmussen et al., 2024) and hence industrial operators need to understand these differences when marketing nanomaterials. The OECD recommended that existing legal frameworks, fine-tuned to nanomaterials, can be used to address the possible risk of nanomaterials (OECD, 2013; 2017c).

• The realization that size at the nanoscale is the only common property of all nanomaterials. However, size alone is not an indication of hazard
• Creation of regulatory definitions of ‘nanomaterial’ and the awareness of their differences for different legislative areas and geographical regions

### 9.2 Testing and data

Based on earlier discussions of “ultrafine particles”, it was acknowledged from the start that nanomaterials have the characteristics of both chemicals and particles. Furthermore, minor modifications of the physico-chemical characteristics may lead to significant changes of the nanomaterial’s functional or ecotoxicological properties, and should hence be carefully considered when addressing nanomaterials’ potential hazards. An initial issue considered was the applicability to nanomaterials of tests developed for chemicals. Especially for ecotoxicology testing, the dose-metrics, and description of the uptake of nanomaterial in organisms, the toxicokinetics, and the fate and behavior of nanomaterials were seen as needing special attention. For example, nanoparticles can be taken up actively by cells and were demonstrated to be able to translocate. Several new OECD TGs for nanomaterials have been developed, and selected existing TG have been adapted to nanomaterials or supported by guidance documents, see Table 1. The sample preparation, including dispersion media, is fundamental for any testing of a nanomaterial, and much effort has been invested in developing dispersion protocols, leading to the OECD GSPD (OECD, 2012), for which an update is expected in 2025. Furthermore, the unique behavior of nanomaterials might give rise to artefacts during testing (Petersen et al., 2014; 2015), which should be avoided.

• The particulate nature of nanomaterials induces new ecotoxicology dimensions compared to soluble chemicals
• The nanomaterials’ safety profile depends on several physico-chemical properties.
• Sample preparation is an important step in nanomaterial testing
• Artefacts during nanomaterial testing should be avoided
• OECD TGs must take into account the particulate nature of nanomaterials throughout all steps from testing to interpretation of the results
• Several new TGs have been developed as a result of this insight

The development of alternative methods for testing nanomaterials has been prioritized and an increasing number of tools and frameworks of alternative methods are available, though internationally recognized methods takes long to endorse; ways of accelerating the recognition of such methods are looked into. A possible nearer-term alternative method for assessing key hazard endpoints such as pulmonary toxicity is through the development of more robust groupings of nanomaterials to enable read-across, interpolation, and the use of IATAs. The OECD is focusing on achieving nanomaterial-relevant genotoxicity protocols. In addition to regulatory accepted *in vitro* methods, research looks into the application of NAMs as a means to early hazard identification even if the regulatory acceptance and uptake of NAMs still need to materialize (Haase et al., 2024; Berggren and Worth, 2023). Despite the progress in developing *in vitro* models, nanospecific methodologies are still very fragmented and there is a general lack of harmonization in, e.g., how to conduct, design and report experiments, the choice of the model to be used, the exposure of the biological system, the dispersion protocols, and the dosimetry (Haase et al., 2024; Cattaneo et al., 2023).

• For example, *in silico, in chemico,* and *in vitro* methods suitable for nanomaterials are needed
• Nanospecific *in vitro* methodologies still need harmonization in e.g., how to conduct, design and report experiments, the choice of the model to be used, the exposure of the biological system, the dispersion protocols, and the dosimetry
• The development of qualified NAMs may be a way forward
• A nearer-term alternative method to obtaining data useful to regulatory authorities may be to further develop groupings of nanomaterials for assessment of hazard toxicological and ecotoxicological endpoints. This may require additional testing, using methods currently acceptable to regulators. Industry and regulatory authorities should collaborate in this effort to ensure the resulting data are acceptable to regulators.

The availability and quality of data is crucial, also to enable grouping of nanomaterials. The importance of FAIR data is recognized and includes detailed reporting of metadata associated to any measurement results (Jeliazkova et al., 2021). FAIR data is supported by the development of OHTs for some nanospecific endpoints (Rasmussen et al., 2019).

Reference nanomaterials is a means of improving data quality, and the availability of representative test materials from the JRC repository was a major step in that direction and improves the nanomaterial safety testing by ensuring a better comparability of nanomaterial test results.

• New data generated must be FAIR and of high quality
• The data comparability and quality is improved by the availability of representative test materials
• Reference nanomaterials are needed

### 9.3 Exposure

For safety assessment of nanomaterials it is fundamental to establish the routes of exposure and quantify nominal and internal exposure in the medium or organism (Behzadi et al., 2017). This requires analytical quantification of the actually internalized fraction of the applied dose of nanoparticles, which continues to be a major challenge.

• For nanomaterials there are still major analytical challenges in determining the internal dose

For environmental exposure, issues include the identification and quantification of (minute) amounts of manufactured nanoparticles, low concentration, hetero- and homo-agglomeration structural heterogeneity, and dynamic transformation of nanomaterials in (complex) environmental matrices (Lowry et al., 2012; Jiang et al., 2022) as well as transport of nanomaterials between environmental compartments. For nanomaterials, the OECD has developed regulatory recognized TGs and GDs for assessing their environmental fate and behavior, see table 1, and evaluated tools and models for assessing environmental exposure (OECD, 2021d). Analytical techniques for identifying and measuring nanomaterials quantitatively in environmental systems have increased and the measurement capability have improved (Jiang et al., 2022). Estimation of exposures to nanomaterials are complicated by nanomaterial transformation in the environment. There are still significant methodological gaps and some GDs would need significant modifications to specifically consider the hazard implications related to the particulate nature of nanomaterials. The development of environmental exposure models for nanomaterials is progressing, noting that field data sets for validation are lacking.

• Though there are still major gaps, the identification and quantification of nanomaterials in complex media is progressing.
• Exposure models are being developed, but there is a need for more cost-effective nanomaterial-specific detection methods to provide field monitoring data for their validation.

Considerations for human exposure to nanomaterials often focus on occupational exposure, and especially inhalation exposure, as this is perceived to have the highest frequency, duration and level of human exposure, and noting that inhaled (nano)particles and (nano)fibers can deposit in the respiratory system. Standards for measuring nanomaterial dustiness have been developed (CEN, 2019a; b; c; d; e) and an OECD TG is under development. However, all routes of exposure (inhalation, dermal and oral) are relevant for workers handling nanomaterials (Basinas et al., 2018). Regarding dermal exposure, *in vitro* studies indicate that the nanoparticles barely pass the stratum corneum (Saweres-Argüelles et al., 2023). *In vivo* studies on uptake of nanoparticles when applying sunscreens indicate that intact skin appears to be a good barrier to prevent systemic exposure, as the nanomaterials were found in the stratum corneum in all cases, but only exceptionally in blood and organs (Saweres-Argüelles et al., 2023).

Efforts have been directed towards modelling nanomaterial exposures and some of the workplace exposure models available for general chemicals have been adapted to nanomaterial exposure. The OECD published an evaluation of 32 identified exposure models/tools for assessing occupational and consumer exposure to manufactured nanomaterials (OECD, 2021a-c), of which 23 models/tools were relevant for workplace exposure.

• Occupational exposure is regarded as the most relevant human exposure to nanomaterials
• Some nanospecific (occupational) exposure models are available.

Our paper presents a case study on production of CNTs that illustrate some of the difficulties the lack of data can create. The reasons for the data gap are multifaceted and include that industry might not be legally required to generate the data and does not volunteer to do it, whereas researchers mostly work at the cutting edge of specialization and do not generate ‘routine’ data, as well as data confidentiality issues. These data should be derived from hazard testing of commercially relevant nanomaterials using accepted OECD TGs such as those for subchronic toxicity testing, and air-monitoring data gathered from occupational settings where nanomaterials are used/produced. Such hazard and exposure data can in turn be used to build more effective groupings of nanomaterials to address regulatory hazards, and to develop more realistic workplace exposure models. Confidentiality issues may be overcome by so-called trusted environments (Soeteman-Hernandez et al., 2019) that would promote the sharing of sensitive information. Furthermore, the (un)availability of regulatory accepted test methods, e.g., OECD TGs, is an important aspect. For inhalation toxicity studies, OECD TGs for sub-acute and sub-chronic exposure durations to nanomaterials are available, whereas adaptation to nanomaterials of the inhalation chronic toxicity is missing. Furthermore, OECD TGs/GDs for testing nanomaterials regarding mammalian biodistribution/biokinetics are still lacking, though a GD for toxicokinetics is under development (see Table 1). Bleeker et al. (2023) give an overview of needs for developing regulatory methods for nanomaterials.

• As industry continues to innovate new nanomaterials, sharing of generated relevant hazard and exposure data that allow for both better read across estimations for hazard, and improved models for estimating exposures in the workplace. This work should be done in conjunction with regulatory agencies to ensure it is directly relevant to current and future commercial scenarios and identification of safe and sustainable solutions.
• The creation of trusted environments, i.e., formal agreements on the frame for data sharing, could promote data sharing with authorities.

### 9.4 Outlook

During the past 25 years, major progress has been achieved in understanding what the additional issues for the safety of nanotechnology and nanomaterials are, including the areas of definition, testing, and estimating exposure, as outlined above. Several issues identified early on have already been addressed. However, some important remaining gaps, such as the unavailability of best dosimetry, alternative test methods, FAIR data, and analytical tools for quantifying nanomaterials in air and other complex environmental matrices, should be addressed. Further, additional pulmonary toxicity studies, and chronic ecotoxicity studies and environmental monitoring studies would be useful to regulatory decision-making.

The policy context for nanomaterials is moving towards a holistic governance approach embracing sustainability dimensions. OECD has published the Safe(r) and Sustainable Innovation Approach (SSIA) for nanotechnology and (relevant) advanced materials, which is a complementary approach that enhance regulatory preparedness and an early (pre-data) dialogue between innovators and regulators (OECD, 2020d; 2022c). Nano risk governance can be strengthened through the availability of TGs and standards applicable to nanomaterials (Rasmussen et al., 2023; Bleeker et al., 2023), which are yet unaddressed for also chemicals (e.g., NAMs), and thus when developing methods for nanotechnology, this greater chemicals picture should be taken into account. In the EU, the Safe and Sustainable by Design (SSbD) concept and associated JRC framework (Caldeira et al., 2022) has been suggested as an innovation approach to achieve the aims of the Chemicals Strategy for Sustainability (CSS; European Commission, 2020) which is part of EU’s Green deal (European Commission, 2019a) and at the same time maintain the EU’s international competitiveness (European Commission, 2025). The CSS underlines that sustainability is the ultimate goal of appropriate risk governance of new technologies and products and promotes the use of one coherent framework for SSbD chemicals across different stakeholders, e.g., industry and policymakers. For nanotechnology, these policies, combined with work in the nanomaterial-research community, move towards governance of nanotechnology and nanomaterials. To support such policy initiatives methodological approaches and decision support tools have been or are being (further) developed, which can also be applied to support nano risk governance (Caldeira et al., 2022; European Commission, 2022b). The NanoSafetyCluster has integrated work on SSbD and published a roadmap towards safe and sustainable advanced and innovative materials (Cassee et al., 2024).

The nanomaterial area is evolving into more advanced materials with additional groups of relevant related materials, such as multicomponent nanomaterials and nanoscale advanced materials. These materials present even further opportunities and challenges for the assessment, and they are already being discussed in research projects to understand if changes to legislation would be needed, and if so which changes (Hunt et al., 2025). When developing such materials, the EU’s new approach to innovation includes the Safe and Sustainable by Design approach, which could ensure that as far as possible such materials would be safe and sustainable from the outset (European Commission, 2022b; Garmendia et al., 2025). Globally, the OECD also promotes development in this direction, which has resulted in a framework on “Safe(r) and Sustainable Innovation Approach” (OECD, 2020c).

## Footnotes

1
https://ec.europa.eu/health/scientific_committees/opinions_layman/en/nanotechnologies/l-3/1-introduction.htm#0p0
